# PW02-029 - Single cell fluorescent immunoassay of CINCA/NOMID

**DOI:** 10.1186/1546-0096-11-S1-A170

**Published:** 2013-11-08

**Authors:** K Nakagawa, N Shimura, Y Shirasaki, M Yamagishi, K Izawa, R Nishikomori, T Kawai, T Yasumi, T Heike, O Ohara

**Affiliations:** 1 Department of Pediatrics, Kyoto University Graduate School of Medicine, Kyoto, Japan; 2 Research Center for Allergy and Immunology, RIKEN, Yokohama, Japan; 3 KAZUSA DNA research institute, Kisarazu, Japan

## Introduction

CINCA syndrome, also known as NOMID, is a rare autoinflammatory disease caused by the *NLRP3* mutations. It has been known that conventional genetic analysis failed to detect disease-causing mutations in approximately 40% of patients. We have recently identified *NLRP3* somatic mosaicism on 70% of these ”mutation-negative” patients in the international collaborative study (Tanaka N. and Izawa K. et al., Arthritis Rheum, 2011), and found no significant differences on systemic inflammation between heterozygous germline mutations and somatic mosaicism. This raises a question how a small number of *NLRP3*-mutated cells cause systemic inflammation as severely as 100% of germline mutations.

## Objectives

To solve the question, we analyzed cytokine production from the single cells, especially IL-1β which is the key molecule of NLRP3 inflammasome. There are the 2 forms of IL-1β, namely preform and mature forms, and only the latter is said to be secreted. Although the IL-1β in the single cell can be measured by intracellular cytokine staining, the relationship of the amount of intracellular IL-1β in the single cells and secreted IL-1β from them is still unknown. From these issues, it would be a better approach to measure the secreted IL-1β at a single cell level.

## Methods

In this work, we tried to establish a single cell fluorescent immunoassay system to measure IL-1β secretion from monocytes of CINCA/NOMID patients at a single cell level using a soft lithographic method called microengraving.

## Results

In the healthy control, very small number of cells secreted low amount of IL-1β by the LPS stimulation. In contrast, significant number of cells secreted high amount of IL-1β by the LPS stimulation in CINCA/NOMID patients with heterozygous germline mutations. We were also able to observe a large number of IL-1β secreting cells from patients with somatic mosaic mutations by LPS stimulation alone.

To delineate whether only the cells with a mutation on *NLRP3* are responsible for IL-1b secretion in the mosaics, we pick up the single cells from the microwells positive on IL-1β secretion by micromanipulator. By performing genetic analysis on them, we are now trying to determine whether only the mutated cells secrete IL-1β or the small fraction of *NLRP3*-mutated cells causes *in vivo* bystander activation of the wild type monocytes in the somatic mosaic CINCA/NOMID patients.

In addition, this method could be offered to diagnose the somatic mosaicism of CINCA/NOMID easily on the basis of single cell functional analysis, which would complement the DNA sequencing based method (Izawa K. and Hijikata A. et al., DNA research, 2012) that might miss some rare CINCA/NOMID cases caused by other than *NLRP3* coding region mutations.

## Disclosure of interest

None declared.

